# Current developments in surface electromyography

**DOI:** 10.55730/1300-0144.5667

**Published:** 2023-03-26

**Authors:** Veysel ALCAN, Murat ZİNNUROĞLU

**Affiliations:** 1 Department of Electrical and Electronics Engineering, Engineering Faculty, Tarsus University, Mersin, Turkiye; 2 Department of Physical Medicine and Rehabilitation, Faculty of Medicine, Gazi University, Ankara, Turkiye

**Keywords:** Surface electromyography, neurophysiology, electrophysiology, kinesiology, biofeedback

## Abstract

**Background/aim:**

Surface electromyography (surface EMG) is a primary technique to detect the electrical activities of muscles through surface electrodes. In recent years, surface EMG applications have grown from conventional fields into new fields. However, there is a gap between the progress in the research of surface EMG and its clinical acceptance, characterized by the translational knowledge and skills in the widespread use of surface EMG among the clinician community. To reduce this gap, it is necessary to translate the updated surface EMG applications and technological advances into clinical research. Therefore, we aimed to present a perspective on recent developments in the application of surface EMG and signal processing methods.

**Materials and methods:**

We conducted this scoping review following the Joanna Briggs Institute (JBI) method. We conducted a general search of PubMed and Web of Science to identify key search terms. Following the search, we uploaded selected articles into Rayyan and removed duplicates. After prescreening 133 titles and abstracts, we assessed 91 full texts according to the inclusion criteria.

**Results:**

We concluded that surface EMG has made innovative technological progress and has research potential for routine clinical applications and a wide range of applications, such as neurophysiology, sports and art performances, biofeedback, physical therapy and rehabilitation, assessment of physical exercises, muscle strength, fatigue, posture and postural control, movement analysis, muscle coordination, motor synergies, modelling, and more. Novel methods have been applied for surface EMG signals in terms of time domain, frequency domain, time–frequency domain, statistical methods, and nonlinear methods.

**Conclusion:**

Translating innovations in surface EMG and signal analysis methods into routine clinical applications can be a helpful tool with a growing and valuable role in muscle activation measurement in clinical practices. Thus, researchers must build many more interfaces that give opportunities for continuing education and research with more contemporary techniques and devices.

## 1. Introduction

Surface electromyography (surface EMG) is a main technique used to detect and analyze the electrical activities produced by skeletal muscles through surface electrodes on the skin. It provides important information about muscle control by the nervous system (central and peripheral). To prevent disorders and evaluate treatments, surface EMG is conventionally used to understand specific conditions, such as muscle fatigue, denervation, reinnervation, muscle coordination, load sharing, and spasticity. In recent years, the applications of surface EMG have extended beyond traditional fields to encompass new areas, including obstetrics, occupational medicine, art in medicine, neuro-rehabilitation, ergonomics, preventive medicine, research on aging, veterinary science, control of artificial limbs, robotics, and the development of human-machine interfaces [1]. Because of this important expansion into new application fields, the number of potential users has also increased.

The rapid development in research fields and cutting-edge technologies are fostering the growth of surface EMG and its potential in clinical applications. Developments in EMG applications can make the use of this technique more attractive for clinicians by taking EMG out of the traditional environment. However, there is still a lack of acceptance of surface EMG in clinical applications. Many challenges remain unresolved, including adaptation to novel EMG systems and signal processing methods [1,2]. These challenges led to a gap in progress between the research fields of surface EMG and its clinical acceptance, characterized by the translational knowledge and skills in the widespread use of surface EMG among the clinician community. To reduce this gap, it is necessary to translate updated surface EMG applications and technological advances into clinical research. However, it can be difficult for the clinicians or clinical practitioners to follow all the aspects of signal processing and technological innovations in surface EMG Therefore, we aimed to present a perspective on recent developments in the application of surface EMG and signal processing methods.

## 2. Materials and methods

We conducted this scoping review following the Joanna Briggs Institute (JBI) method and included studies that focused on surface EMG and its applications. Based on the JBI recommendations, we conducted a general search of PubMed and Web of Science to identify key search terms. Our search string was: (“Surface Electromyography” OR “Surface EMG” OR “sEMG” OR “Electromyography”) AND (“Kinesiology” OR Biofeedback” OR “Neurophysiology” or “Muscle Strength” OR “Muscle Fatigue” OR “Physical Therapy and Rehabilitation” OR “Physical Exercise” OR “Posture” OR “Postural Control” OR “Movement Analysis” OR “Muscle Coordination” OR “Motor Synergies” OR “Modelling” OR “Signal Processing” OR Signal Analyzing” OR “Decomposition”) NOT (“review”[Title/Abstract] OR “Needle Electromyography” OR “needle EMG”). The search string was further expanded by running searches with medical subject headings (MeSH) in the medical databases (e.g., PubMed) as well as with identical and compatible non-MeSH terms in other databases (e.g., Web of Science and Google Scholar). We tested various combinations of search terms with Boolean operators to assess the sensitivity of the terms for word variations. Following the search, we uploaded selected articles into the Rayyan web software and removed duplicates. We did a prescreening of 133 titles and abstracts and assessed 91 full texts of selected abstracts and titles according to the inclusion criteria. We then extracted data from the studies using the JBI guidelines. We conducted a thematic analysis to clarify the conceptual categories. This approach allowed us to characterize the application fields of surface EMG and to assess its clinical acceptance.

## 3. Results

The present study reviewed the latest developments in surface EMG recording, signal analysis, and its application fields.

### 3.1. Surface electromyography detection

**Figure 1:**
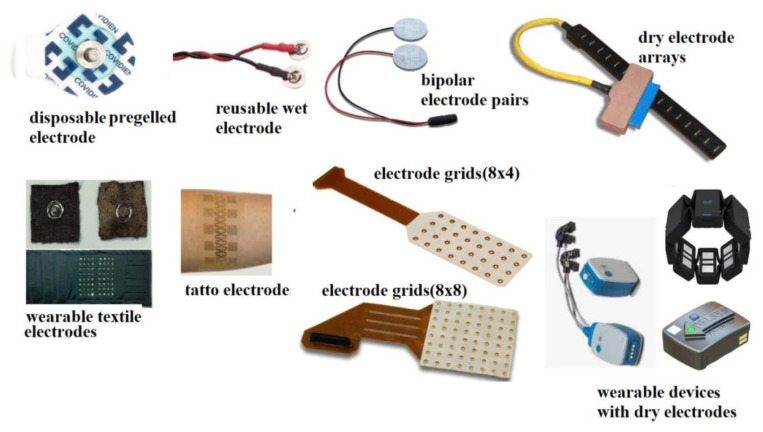
Different surface EMG electrodes.

The source of surface EMG signals is the depolarizing and repolarizing regions of muscle fibers. Motion generation is accomplished by transmitting synaptic inputs to motor neuron pools. When a motor neuron is discharged, action potentials are generated at the neuromuscular junctions and propagated across all muscle fibers to the tendon sites. Motor unit (MU) action potentials are the sum of MUs and can be recorded from the skin surface at various distances from the source by surface EMG. The recording of surface EMG signals includes the following steps: (i) detection of myoelectric potentials with surface electrodes (bipolar electrode pairs, electrode arrays, or grids), (ii) amplification of these potentials, (iii) analog filtering of amplified potentials to prevent aliasing, and (iv) converting analog to digital signals by sampling. Surface EMG signals can be transmitted to computer, tablet, and mobile devices through wired (electrical or optical) or wireless (WiFi, Bluetooth, NFC) connections. Surface electrodes can have different properties and configurations, as shown in Figure 1. These electrodes can be mainly divided into wet (metal–skin contact with a gel or paste), dry (metal–skin contact without gel or paste), and capacitive (no electrical contact with the skin) electrodes [3]. The wet silver/silver chloride (Ag/AgCl) electrode pairs are still widely used as conventional surface EMG electrodes.

Further techniques for detecting surface EMG signals are based on multichannel detection through one- or two-dimensional electrode arrays, which allow the application of spatial filters with different spatial selectivity. One of the important developments in surface EMG devices is high-density surface EMG (HDsEMG), which is EMG imaging technology to identify the recruitment of more than a single MU. However, using HDsEMG in clinical practice is more complex than using conventional surface electrode pairs because of its specific hardware and analysis method [3,4]. Advances in electrode technology continue in parallel with developments in materials science and sensor technology. Research has been conducted on various innovative electrodes, such as nanomaterial-based electrodes, tattoo-like or skin-printed conductive inks, semipermanent tattoo electrodes, high adhesion stretchable electrodes, wearable high-resolution facial arrays, hydrogel electrodes, and anal and vaginal probes with special geometries [5–7].

### 3.2. Factors affecting surface EMG signal characteristics

The signal characteristics of surface EMG depend on several anatomical, physical, and detecting methods. The signal detection can vary according to the size and shape of the electrodes (i.e. electrode pairs, grid electrodes), the electrode material, the interelectrode distance (IED), and the electrode–skin contact (i.e., dry or gelled) [8–10]. Anatomical attributes mainly depend on muscles type and form [8]. For example, a small grid of electrodes with short IEDs (2.5 mm to 5.0 mm) could be well suited for the hand and facial muscles, while the mapping of surface EMG in larger volume muscles such as the calf requires larger electrode grids with larger IEDs (5.0 mm to 8.0 mm). Other anatomical factors are the thickness of the subcutaneous tissue layer, the depth of the source within the muscle, and the length of the muscle fibers [10].

**Figure 2:**
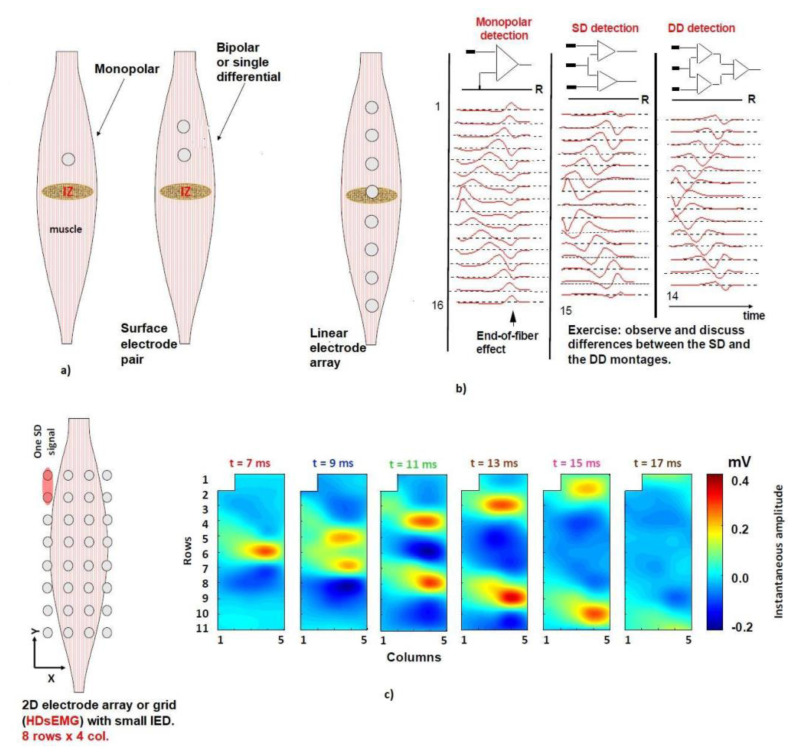
IZ detection with different electrode locations: a) surface electrode pairs, b) linear electrode array, and c) grid electrodes.

The main physical factors are the orientation of the electrodes according to the fiber direction and their position to the muscle’s innervation zone (IZ) region, the slope of the electrodes according to the orientation of the muscular system, the IED, the conductive gel, and the crosstalk [11]. Figure 2 shows that two lines intersecting is a good estimate for the location of the center of the IZ. Figure 2a shows electrode pairs set to reidentify the IZ for more accurate localization of the MUs. While linear electrode arrays allow for precise mapping of MU activity along the muscle length (Figure 2b), grid electrodes allow a comprehensive understanding of how MUs are distributed within the muscle volume (Figure 2c).

The factors related to the detection system are the spatial filters, amplifiers, electrode type, electrode configuration, electrode material, and noise. Noise can occur from electrode–skin contact, the input impedance of the bioelectric signal amplifier, a motion artifact, power line interference, or electromagnetic radiation. The inheritance of the task (dynamic, static, maximal, submaximal, etc.) is one of the essential factors. The effect of all these factors on surface EMG features has been extensively studied and discussed in the literature [11,12].

### 3.3. Signal processing methods for surface EMG

**Figure 3:**
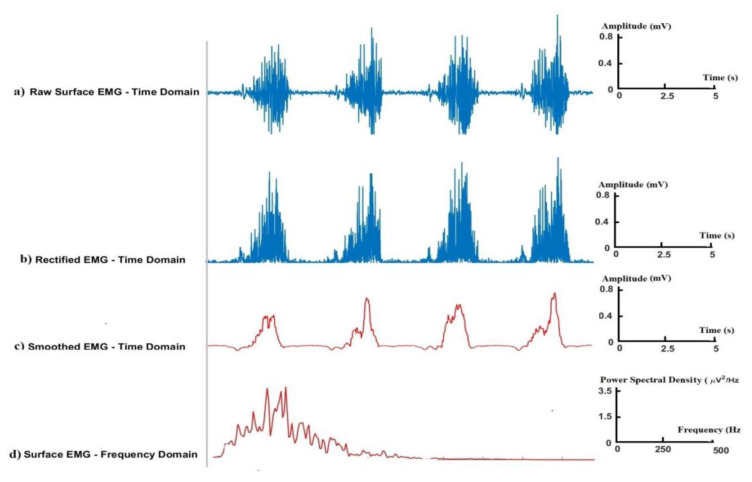
Conventional signal analysis methods for surface EMG: a) raw surface EMG in the time domain, b) rectified surface EMG signal, c) smoothed surface EMG signal, and d) PS of surface EMG signal in the frequency domain.

Signal analysis methods can provide descriptive information and features of the signal. Surface EMG signals have been extensively analyzed in the time domain, frequency domain, and time–frequency domain. Conventional signal analysis methods for surface EMG are shown in Figure 3. These analysis methods mainly involve the examination of the raw surface EMG in the time domain (Figure 3a), rectified surface EMG signals (Figure 3b), smoothed surface EMG signals (Figure 3c), and the power spectrum (PS) of surface EMG signals in the frequency domain (Figure 3d).

EMG features have been recently obtained from both linear and nonlinear analysis methods. The main surface EMG features in the time domain are average rectified values, mean absolute values (MAV), and root-mean-square values (RMS), in terms of amplitude [13]. Many other surface EMG features have been introduced in the time domain by providing different information (energy, frequency, complexity estimation model, etc.). For example, modified mean absolute value (mMAV), average absolute value slope (MAVSLP), variance (VAR), integrated EMG, v-order, LOG detector, and simple square integral variables. Absolute temporal moments provide statistical information similar to the MAV and VAR properties. Some features provide frequency information of the EMG signal defined in the time domain, such as zero crossings (ZC), Willison amplitude (WAMP), myopulse percentage rate, slope sign change, and amplitude of the first burst. A histogram is an extension of the ZC and WAMP features. The waveform length (WL) provides information about the complexity of the EMG signal in the time domain. Change in average amplitude and the difference of the absolute standard deviation are different versions derived from the properties of WL. Autoregressive coefficients provide information for the prediction model. Multiple Hamming windows and multiple trapezoidal windows provide energy and complexity of signal information with the windowing method [13–15].

The signal analysis in the frequency domain provides useful and important spectral features of signals that cannot be obtained in the time domain. For transforming the signal into the frequency domain, the PS or power spectral density (PSD) is calculated using a Fourier transform of the autocorrelation function of the EMG signal. All features in the frequency domain are calculated according to the statistical parameters of the PSD. The most common features of surface EMG signals in the frequency domain are mean frequency (MNF) and median frequency (MDF). Modified MNF and modified MDF were proposed as expanded versions of MNF and MDF. Other features can be mathematically obtained from the EMG PS, such as total power, mean power, peak frequency (PKF), and frequency ratio (FR). Spectral moment and central frequency variance are alternative forms of statistical analysis calculated from the EMG PS. PS ratio extends the PKF and FR features. Cepstral coefficients are calculated as the inverse Fourier transform of the signal’s logarithm of the PS size. The PS distortion rate (Ω) provides information about spectral distortion. SNR is the ratio of signal power to noise power [16,17].

The time–frequency analysis provides information about the frequency spectrum in a fixed time interval or where the components of the signal in a certain frequency range are at a certain time. This method has attracted the attention of many researchers. The most common methods in the time-frequency domain are the Wigner–Ville distribution, the Choi–Williams distribution, short-time Fourier transform, wavelet transform, empirical mode decomposition, and the Stockwell transform [18–20].

Statistical methods such as higher order statistics, independent component analysis (ICA), skewness, and kurtosis are being used in surface EMG analysis. Different ICA algorithms are also used in surface EMG signal analysis [21]. To explain the distribution of sampled EMG signals, the probability density function basis of probability theory is used [22]. Nonlinear time series analyses are used to investigate the surface EMG signal in terms of stochastic, deterministic, and even chaotic characteristics. These nonlinear methods include the surrogate data method, the Volterra–Wiener–Korenberg model, chaotic analysis method, symplectic geometry method, correlation, fractal analysis method, and entropy [23,24].

HDsEMG represents two-dimensional (2D) potential distribution that provides an image of an instantaneous surface EMG for each sample. Image interpolation has been proposed to reduce the pixel size and create additional smaller pixels for a smoother display of HDsEMG with appropriate sampling. It can sample this 2D analog signal by sequencing the maps in space and time. Each epoch provides an image of the spatial distribution of the MAV, RMS, MNF or MDF features. It can also create a surface EMG analog movie with frames separated by epoch duration and colors representing the instant potential amplitude distribution in time.

### 3.4. Application fields of surface EMG

The review results show that surface EMG has had a wide range of applications as a muscle activation measurement tool that can transfer information about muscle function. The table shows the different application fields of surface EMG.

Neurophysiology is one of the main application fields of surface EMG for assessing muscle activation, neural activation patterns, and movement strategies in humans [25–27]. The use of surface EMG has progressively expanded with the enhanced knowledge of the physiopathology of disease, the diagnosis of the neuromuscular diseases, and evaluation of patient rehabilitation and treatments [28–31]. Surface EMG has been extensively investigated to evaluate the effects of strengthening training programs, muscle activation, neural adaptation, the plasticity of the nervous system following exercise training, aging factors in the muscle and nervous system, and the neural regulation required to perform different motor tasks [28–31]. Electrical stimulation methods have also been used in combination with surface EMG. For example, they are used together for estimating myoelectric signal variables, evaluating muscle activation in exercise training, understanding the functional control of paralyzed extremities, and rehabilitating stroke, spinal cord injury, geriatric, and cardiovascular patients [32–34]. The evoked potentials of electrical and magnetic stimulation (M wave, H-reflex, MEP, and CMEP, etc.) have been conducted to investigate neuromuscular fatigue and the provocation of the corticospinal system during different motor tasks. EMG response to nerve stimulation has been used to investigate the number of active motor neurons, evaluate denervation, and monitor neurodegenerative processes [35].

To understand the mechanical and myoelectric events of muscle fatigue and muscle strength during various contractions, surface EMG has been studied in the fields of sports, rehabilitation, occupational medicine, space medicine, prosthetic control, work-related disorders, and oncology [2,36,37]. Joint torque measurements have been used in isometric contractions in many clinical applications, such as for stroke, Parkinson’s disease, multiple sclerosis, diabetes, and other various pathologies [38,39].

In physical therapy applications, surface EMG has been commonly used to evaluate muscle activity during tasks such as postural perturbations, the myoelectric signs of muscle fatigue, and the magnitude of muscle activation [40–42]. It has also been applied to evaluate impaired motor control in physical therapy and to monitor the changes by rehabilitation [43,44].

Posture and gait studies use surface EMG to investigate the role of muscles and the mechanisms of reflex and control paradigms [45–47]. Many studies have examined EMG responses for postural disorders, activation of muscles, internal sources of postural perturbations, and changes in dementia translations [48–52]. Surface EMG provides qualitative and quantitative information (timing, amplitude, and morphology of muscle activation) for many applications (e.g., clinical evaluation, surgical selection, local muscle inhibition by drugs, assessment of muscle strength, muscle fatigue, orthoses, masticatory functions, dental malocclusions, and rehabilitation protocols) [53–56].

To explore neural control, assessment of muscle coordination and motor synergy is also one of the application fields of surface EMG. Measuring muscle activity during motor behavior helps to estimate the net global firing rate of spinal motor neurons innervated by muscle and envelope changes [57,58]. Therefore, surface EMG provides a quantitative perspective in neurophysiology research to examine the complex control mechanisms of the central nervous system, which drives hundreds of muscles to control whole-body movements. Different approaches have been proposed to reveal the modular motor organization of human behavior, such as muscle synergies in response to postural perturbations, reaching out and movement with the arm, and assessment of the plasticity and flexibility in movement disorders [59–62]. Furthermore, different muscle synergy models and patterns in the organization of the musculoskeletal system have been used to investigate the dynamic behavior of the muscles [63,64]. Surface EMG can characterize the mechanisms underlying muscle coordination, considering the spatial size and temporal nature of the spinal motor output [65]. Using different statistical and analytical approaches, surface EMG studies have revealed a large part of muscle synergy, modulation, and variation in muscle activity between tasks (e.g., standing, balance, and posture control during walking) [65,66]. Besides, many studies have shown different movement modes, directions, speeds, and basic patterns during body support through the phasic and tonic components of surface EMG. Surface EMG has also been applied to different populations, from infants to the elderly, to characterize the specific features of motor pool activity during the development of locomotor patterns and the specific adaptation of segmental motor output in patients [67].

Surface EMG biofeedback has an important role in pathological conditions to provide the patient with advanced information about muscle activity. For example, it benefits both patient and practitioner to learn the locations of muscle tension in the body, how to relax muscles, and the effectiveness of exercises. When combined with other physiotherapies, surface EMG biofeedback has provided better improvements in motor strength, functional recovery, gait quality, and swallowing difficulties compared to standard physiotherapy. Many studies have reported surface EMG biofeedback’s effectiveness in the treatment of patients with stroke, cerebral palsy, and spinal cord injury. It is a beneficial tool for reducing the excitability of hyperactive spinal stretch reflexes. It also improves surface EMG amplitude and various gait parameters (walking speed, cadence) and facilitates the activation of targeted muscles [68–70]. Surface EMG biofeedback training has also effectively treated headaches, asthma, muscle cramps, and pain [71]. To treat incontinence with surface EMG biofeedback, practitioners have used different EMG sensors for detecting and monitoring muscle tension. Several studies have confirmed an association between pelvic muscle dysfunction and vulvar pain symptoms, the muscle spasm-based diagnostic for vaginismus, interstitial cystitis, and urinary incontinence [72]. Surface EMG biofeedback training of masticatory muscles has been used as an effective treatment for temporomandibular disorders when combined with adjunctive cognitive-behavioral therapy [73,74]. Surface EMG biofeedback has been used for motor learning in sports, recreation, and rehabilitation to improve the learner’s performance [75].

Multichannel EMG or HDsEMG explores motor control strategies and motor adaptation to various conditions (pathology, fatigue, pain, or exercise) by decomposition of MUs [76,77]. The automatic decomposition algorithms of MUs have allowed the identification of full MU discharge patterns even at maximum contraction forces. This method has been used to assess neurodegenerative diseases, stroke, type II diabetes, and cleft lip patients [78,79]. Recently, surface EMG simulators have supported the realistic simulation of complex muscle features such as changes in tissue conductivity, the effects of fatigue, and anatomical variations [80]. In addition, advanced simulators of muscle control strategies have been proposed for both healthy and pathological conditions. Surface EMG is a valuable tool in modelling and understanding muscle function and performance, such as the estimation of muscle fatigue, crosstalk, muscle dynamics, deep muscle activation, motor constraints, and joint moments [81–87].

In ergonomics and occupational medicine, surface EMG is an important tool to provide the quantitative evaluation to analyze the neuromuscular system in the work environment and identify risk factors in terms of work-related health problems, such as manual lifting tasks, workloads, posture, horizontal distance, the site of the spine subjected to force, and muscle fatigue [88–90]. Therefore, surface EMG techniques have been extensively used in designing and analyzing workplace optimization, performing arts, the use of musical instruments, risk prevention, and early detection of work-related health problems. Surface EMG has further applications in proctology and obstetrics [91,92]. It is often used in conjunction with other diagnostic and monitoring methods, such as clinical examinations, ultrasound, and pressure sensors. In obstetrics, it is more commonly use to observe the pelvic floor and uterine contractions rather than fetal monitoring, which is typically done using a fetal heart rate monitor and ultrasound.

In sport science and exercise physiology, surface EMG has been used to evaluate the effectiveness of exercise training, to investigate the links between coordination and fatigue, and for motion analysis. The main applications in these fields are: (i) evaluating muscle coordination and muscle activity during complex movement patterns (e.g., walking, running, cycling, golf, tai chi, etc.), (ii) observing neural and hypertrophic factors of muscle strength gain, muscle fatigue, and muscle damage, (iii) specifying goal-oriented physical exercise, and (iv) determining suitable exercise for a particular training purpose [93–95].

Surface EMG has been used for other specific applications, such as human–machine interfaces, data-driven or model-driven approaches for prostheses, orthoses, electrical stimulation systems, replacements, orthotics, neuromodulator robots, and robotic assistive devices [96–99]. In neurorehabilitation, the control signals of external devices have been extracted from anatomical and physiological information related to musculoskeletal power generation.

### 3.5. Future directions of surface EMG

Surface EMG has excellent potential in many exciting and growing fields, as shown by the large amount of published literature (20+ books, 200+ reviews, 20+ encyclopedia entries, and 7000+ articles) and the abundance of professional meetings and networking (e.g., the Association for Applied Psychophysiology and Biofeedback, the Biofeedback Foundation of Europe, and the International Society of Electrophysiological Kinesiology, etc.). Similarly, there are rapid advances in equipment and devices provided by surface EMG equipment manufacturers (TMSI, OTbioTech, Delsys, Noraxon, Thought Technology, and others). The current trends include integrated surface EMG and inertial measurement units. The recent launch of wearable EMG motion control devices, such as the Myo armband (http://www.myo.com), is an important indicator of these developments. These advances in wearable technologies have increased the potential of myoelectric devices to penetrate daily life. Future applications may also include using a large array of electrodes that cover all limbs with signal conditioning and wireless transmission embedded in a microcard. The advances in surface EMG biofeedback technique can provide tools to evaluate gross motor activities during various tasks. HDsEMG devices with wireless systems are also rapidly developing. The development of HDsEMG decomposition algorithms will probably focus on online MU identification and feedback for clinical practice and myoelectric control systems. Furthermore, EMG imaging techniques can potentially expand in the future as a solution to the technological problems of the electrode–skin interface. Dry or capacitive wearable electrodes that do not require skin preparation have already been used in telemedicine, sports, and telerehabilitation. Recent developments in surface EMG imaging combined with ultrasound or functional magnetic resonance imaging can play an important role in physiological research. Surface EMG images have the potential to be the input of human–machine interfaces and rehabilitation robots. The studies in this field are interdisciplinary and cover the fields of chemistry, dermatology, textiles, leather-like electronics, organic semiconductors, and materials science. Recent advances in information and communication technology, such as the internet of things, allow for improved communication with medical doctors or biofeedback practitioners in daily life. Therefore, novel technological developments in motor control and motor learning can bring new solutions for surface EMG biofeedback and telerehabilitation applications that lead to more effective physical therapy in many applications.

## 4. Conclusion

It is apparent that surface EMG has made innovative technological progress and has great research potential. Translating these innovations into routine clinical applications can allow them to play a growing and valuable role in muscle activation measurement in clinical practices. Thus, researchers must build interfaces that give opportunities for continuing education and research with more contemporary techniques and devices. These interfaces can support the widespread use of surface EMG in clinical practice.

## Figures and Tables

**Table:** The application of surface EMG across a variety of disciplines.

Field	surface EMG applications
Neurophysiology	spasticity, cramps, and related phenomena [19,25]
	effects of strength training on muscle activation [26]
	gait and stance analysis [27]
	the effects of aging on the muscle and nervous system [28,29]
	evoked potentials (CMAP, M wave, H-reflex, MEP, and CMEP) stimulated by electrical and magnetic stimulation [30]
	muscle training in sports [31]
	functional control of paralyzed extremities [32]
	diagnosis and rehabilitation of neuromuscular diseases (stroke, cerebral palsy, spinal cord injury, etc.) [33,34]
	monitoring the neurodegenerative processes of motor neuron diseases [35]
Muscle strength and fatigue; physical therapy and rehabilitation	the mechanical and myoelectric signs of muscle fatigue [36]
	muscle fatigue in central and peripheral nervous system disorders [37]
	joint torque in isometric contraction in various pathologies [38,39]
	evaluating muscle activation during a task [40,41]
	tuning the curves of the surface EMG amplitude [42]
	monitoring the physiological efficacy and changes of rehabilitation [43,44]
Posture and movement analysis	the role of muscles in postural control [45]
	reflex and control paradigm [46,47]
	muscle activation for postural disturbances (perturbation directions) [48]
	changes in EMG responses in dementia translations [49]
	internal sources of postural perturbations [50]
	the role of the mechanisms and different skeletal muscles involved in the control of the perturbation and quiet stance [51,52]
	analyzing movement and movement disorders [53,54]
	evaluating the effectiveness of applied treatments [55]
	examining jaw, tongue, and cheek movements [56]
	envelope studies [57]
Muscle coordination and motor synergies	spinal motor neuron estimates [58]
	investigating muscle synergies in response to postural perturbation and posture control [59,60]
	modular control of reach movements [61]
	analysis of behavior such as plasticity and flexibility in movement disorders [62]
	factorization to muscle synergies [63]
	reconstruction of spine maps from surface EMG [64]
	motor principles in human walking [65]
	bilateral coordination [66]
	development of locomotor primitives [67]
Biofeedback	the treatment of patients with stroke, cerebral palsy, and spinal cord injury [68–70]
	the treatment of neck and headaches [71]
	the treatment of urinary incontinence [72]
	the treatment of chewing disorder [73]
	dysphagia therapy [74]
	neuromotor rehabilitation and exercise programs [75]
Decomposition	motor control strategies [76]
	morphological and functional properties of MU [77]
	examination of MU under conditions (pathology, fatigue, pain, or exercise) [78,79]
	surface EMG simulation [80]
Modeling	muscle fatigue modeling [81]
	understanding crosstalk [82]
	estimating muscle dynamics reflecting joint moments [83]
	investigating motor constraints in pathological conditions [84,85]
	examining deep muscles [86]
	estimating muscle stimulation, muscle–tendon unit strength, and joint moment [87]
Other applications	ergonomics (ergonomic design and analysis to prevent work-related disease and early detection of musculoskeletal disorders) [88–90]
	proctology and obstetrics applications [91,92]
	sports and movement sciences (biomechanics and motion analysis, strength or endurance training, coordination, and fatigue) [93–95]
	rehabilitation technologies and human-machine interfaces (prostheses, orthoses, electrical stimulation systems, control signals to external devices in neurorehabilitation, etc.) [96–99]
